# Expression of CTRP3, a Novel Adipokine, in Rats at Different Pathogenic Stages of Type 2 Diabetes Mellitus and the Impacts of GLP-1 Receptor Agonist on It

**DOI:** 10.1155/2014/398518

**Published:** 2014-08-11

**Authors:** Xin Li, Li Jiang, Miao Yang, Yu-wen Wu, Su-xin Sun, Jia-zhong Sun

**Affiliations:** ^1^Department of Endocrinology, Zhongnan Hospital, Wuhan University, Wuhan 430071, China; ^2^Department of Internal Medicine, Zhongnan Hospital, Wuhan University, Wuhan 430071, China

## Abstract

This study aimed to investigate the expression of C1q/TNF-related protein-3 (CTRP3) in rats at different pathogenic stages of type 2 diabetes mellitus (T2DM) and the impacts of glucagon-like peptide-1 (GLP-1) receptor agonist on it. Male wistar rats were fed with high-fat diet for 10 weeks to induce insulin resistance (IR) and then were given low-dose streptozotocin (STZ) intraperitoneal injection to induce T2DM. Exendin-4 (Ex-4), a GLP-1 receptor agonist, was subcutaneous injected to the IR rats and T2DM rats for 4 weeks. The expression of CTRP3 mRNA and protein in epididymis adipose tissue of rats at the stage of IR was lower significantly than that of normal control (NC) rats and decreased more when they were at the stage of overt T2DM (all *P* < 0.05 or *P* < 0.01). After the treatment with Ex-4, the mRNA and protein expressions of CTRP3 were increased by 15.5% (*P* < 0.01) and 14.8% (*P* < 0.05), respectively, in IR rats and increased by 20.6% (*P* < 0.01) and 16.5% (*P* < 0.05), respectively, in T2DM rats. Overall, this study found that the expression of CTRP3 in visceral adipose tissue was progressively decreased in a T2DM rat model from the pathogenic stage of IR to overt diabetes, while Ex-4 treatment increased its expression in such animals.

## 1. Introduction

Type 2 diabetes mellitus (T2DM) is a chronic disease characterized by insulin resistance and pancreatic islet beta cells dysfunction. The T2DM pathogenesis represents the combined effects of genetics, nutrition, and lifestyle and involves both gene-gene and gene-environment interactions [1]. Over the past few decades, the correlation between the prevalence of T2DM and obesity has been well demonstrated, as more than 80% of T2DM patients are overweight or obese [2]. It is now well known that white adipose tissue is not only serving as a long-term energy store but also as an active endocrine organ that secretes a number of bioactive molecules called adipokines [3]. In addition to being important regulators of adipose tissue development and function, adipokines also have significant impacts on glucose metabolism in various tissues [4]. Abnormal expression and secretion of some adipokines in adiposity are strongly associated with the development and progression of insulin resistance and pancreatic islet beta cell dysfunction, which gradually and ultimately lead to the pathogenesis of T2DM.

C1q/TNF-related protein-3 (CTRP3) is a novel adipokine with multiple effects as lowering glucose levels, inhibiting the glyconeogenesis in liver, and increasing angiogenesis and anti-inflammation. Studies* in vivo* indicated that a modest 3-fold elevation of plasma CTRP3 levels by recombinant protein administration in normal and insulin resistant *ob*/*ob* mice is sufficient to lower glucose levels which may be mediated by up-regulating the protein kinase B (PKB) and inhibiting the expression of the gluconeogenic enzymes glucose-6-phosphatase and phosphoenolpyruvate carboxykinase in liver [5]. A recent study showed that CTRP3 attenuated diet-induced hepatic steatosis by regulating triglyceride metabolism [6], indicating that CTRP3 may be an important regulator of lipid metabolism as well as glucose metabolism.

CTRP-3 is expressed in subcutaneous and visceral adipocytes and is positively regulated by insulin and negatively regulated by chronic lipopolysaccharide (LPS) exposure [7]. One clinical research showed that circulating CTRP-3 concentrations were elevated in patients with T2DM and metabolic syndrome (MS) [8], while another one reported that serum and omental adipose tissue CTRP3 were lower in women with polycystic ovary syndrome (PCOS) and metformin treatment increased serum CTRP3 levels in these women and in omental adipose tissue explants [9]. A study from Korea showed that a 3-month combined exercise program significantly decreased CTRP-3 levels in obese Korean women [10]. Although there has been some researches concerned the expression of CTRP3 and its modulation, little is known about its expression pattern at different stages of T2DM which is a chronic and progressive disease.

Glucagon-like peptide-1 (GLP-1) receptor agonists are a new class of pharmacological agents that improve glucose homeostasis in many ways, including potentiation of glucose-stimulated insulin secretion, glucose-dependent inhibition of glucagon secretion, and reduction in gastric emptying, appetite, food intake, and body weight [11]. Exendin-4 (Ex-4), a GLP-1 receptor agonist that has been used as a drug injected subcutaneously for treatment of T2DM, was shown to promote adiponectin secretion via the protein kinase A (PKA) pathway in 3T3-L1 adipocytes and ameliorate insulin resistance [12]. But it is unknown whether Ex-4 might modulate the expression of CTRP3 in rats with T2DM. So, in this report, we showed the gene and protein expression of CTRP3 in visceral adipose tissue of rats at different stages of T2DM pathogenesis and the effects of Ex-4 on it.

## 2. Research Design and Methods

### 2.1. Animal Feeding and Treatments

Seventy healthy male wistar rats, 8 weeks of age, were placed in a room with controlled lighting (12 hours light/dark cycle) and regulated temperature (18–25°C) and humidity. All rats were fed with regular chow (protein 21%, carbohydrate 55%, fat 6%, and total energy 15.36 kJ/g) for 2 weeks to be adapted for the environment. Twenty-four rats were randomly selected as normal control group (NC) and fed with regular chow throughout the study. The expression of CTRP3 in rats of NC group was detected at the beginning of the study (marked as week 0), week 10 and week 15, respectively, and each of the detection sacrificed 8 rats. The remaining 36 rats were fed with high-fat diet which consisted of regular feedstuff, sucrose, lard, fresh egg, and milk power (protein 16%, carbohydrate 38%, fat 46%, and total energy 20.54 KJ/g) according to one of our former studies [13]. After 10 weeks high-fat diet feeding, twenty-four rats were selected randomly as insulin resistance group (IR, *n* = 16) and IR + Ex-4 group (*n* = 8) which was given 10 *μ*g/kg Ex-4 (ChemPep) administered by subcutaneous injection daily for 4 weeks. The expression of CTPR3 in rats of IR group was detected at week 10 and week 15, respectively, and each of the detection also sacrificed 8 rats. The other 22 high-fat feeding rats were given an intraperitoneal injection of streptozotocin (STZ, 25 mg/kg) in 0.1 mol/L citrate-buffered saline. One week later, random blood glucose of the rats were detected and rats with randomized blood glucose ≥16.7 mmol/L twice not in one day were considered as diabetic ones. There were 15 T2DM rats who were divided randomly into DM group (DM, *n* = 7) and DM + EX-4 intervention group (DM + Ex-4, *n* = 8) treated as IR + Ex-4 group. The control rats received daily saline injections. The expressions of CTRP3 in rats of IR + Ex-4 group, DM group and DM + Ex-4 group were also detected. The body weight of all rats was measured weekly during the study.

### 2.2. Hyperinsulinemic Euglycemic Clamp

Rats of NC group, IR group, DM group, DM + Ex-4 group, and IR + Ex-4 group at week 15 were anesthetized with pentobarbitone (80 mg/kg) after overnight fasting. Both sides of femoral veins were exposed and inserted by a catheter for infusion of glucose and insulin, respectively. Another catheter was inserted into the femoral artery for blood sampling. Rats were kept quiet for 30 minutes, then a 120 minute hyperinsulinemic euglycemic clamp was performed. Insulin was infused at a constant rate of 1.67 mU/kg per minute and the arterial blood glucose concentration was clamped at the basal fasting level by infusing glucose at variable rates. Under the hyperinsulinemic conditions, the steady glucose infusion rate (GIR) required to maintain euglycemia is a standard measure of the whole-body insulin sensitivity.

### 2.3. Real-Time Polymerase Chain Reaction (RT-PCR)

Fresh epididymis adipose tissue specimens were homogenised in RLT lysis buffer (Rneasy Mini Kit, QIAGEN, Valencia, CA, USA) using a rotator-stator, followed by a chloroform delipidation step. The upper aqueous phase was processed for total RNA extraction using silica-based spin columns (Rneasy Mini Kit). The cDNAs were reverse-transcribed with SuperScript III (Invitrogen) from 1 *μ*g of total RNA according to the manufacturer's protocol. PCR primers (Saibaisheng, Shanghai, China) used in the study were as follows: (1) CTRP3 sense 5-GCC CCC GTA TCA GGT GTG TAT TT-3; antisense: 5-TGA AGA CTG TGT TGC CGT TGT GC-3; (2) *β*-actin: sense 5-ACA CCC GCC ACC AGT TCG C-3; antisense 5-TCT CCC CCT CAT CAC CCA CAT-3. The *β*-actin mRNA level was quantified as an internal control. The Δ(ΔCt) method was used to calculate the results for each control and experimental group. The cycle conditions were 50°C for 2 min followed by 40 cycles at 95°C for 15 s and 65°C for 34 s.

### 2.4. Western-Blot

Rats epididymis adipose tissue protein homogenates were prepared in tissue protein extraction buffer (Thermo Scientific) supplemented with protease inhibitors (Roche Applied Science) and phosphatase inhibitors (Sigma). Samples were boiled for 5 min in SDS loading buffer and equal amounts (25–50 *μ*g per sample) of protein extracts were then separated by 8–12% of SDS-PAGE and electrotransferred onto PVDF membrane (Bio-Rad). Membranes were blocked with 5% non-fat skim milk in Tris-buffered saline/0.1% Tween20 (TBS-T) for 1 h, and then incubated with affinity-purified goat polyclonal primary antibodies were used at the following working dilutions: CTRP3 (1 : 1000 dilution, Abcam) and beta-actin: (1 : 1000 dilution, Santa Cruz). Appropriate secondary antibodies conjugated to horseradish peroxidase were incubated with respective membranes for 1 h at room temperature. Following five times intermittent washes with 1 × TBS-T, the membranes was processed for autoradiography using enhanced chemiluminescence (ECL, Pierce Chemical). The results were quantified by densitometric analysis using the Image-Quant software. All Western-blot experiments were performed in triplicate.

### 2.5. Statistical Analysis

Data were expressed as mean ± SE and were evaluated statistically using One-way ANOVA with SPSS (version 19.0) software. A value of *P* < 0.05 was considered to be statistically significant.

## 3. Results

### 3.1. Animal Model

**Figure 1:**
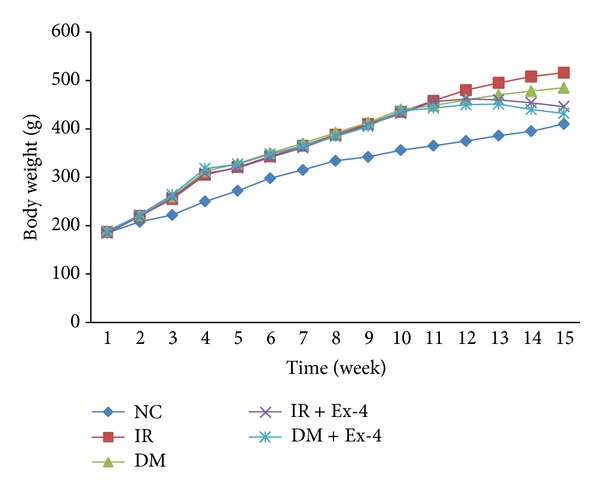
The curve of rats' body weight. NC: normal control group; IR: insulin resistance group; DM: diabetes mellitus group; IR + Ex-4: insulin resistance plus Exendin-4 treatment group; DM + Ex-4: diabetes mellitus plus Exendin-4 treatment group.

The body weight of rats throughout study was shown in Figure 1. At the beginning of the study (week 0), there were no significant differences in body weight among the groups. After high-fat diet feeding, the body weight of rats in IR group, DM group, IR + Ex-4 group, and DM + Ex-4 group increased faster than NC group. There were no significant differences in body weight among IR group, DM group, IR + Ex-4 group, and DM + Ex-4 group before the treatment of Ex-4. At the end of the study (week 15) the body weight of rats in IR + Ex-4 group was lower than that of IR group (*P* < 0.01), and DM + Ex-4 group was lower than DM group statistically (*P* < 0.01). After the STZ intraperitoneal injection in 22 high-fat diet feeding rats, there were 15 rats whose random glucose ≥16.7 mmol/L twice not in one day, being considered as T2DM. Not surprisingly, the fasting blood glucose (FPG) in rats of DM group and DM + Ex-4 group was higher than that of NC group or IR group significantly (all *P* < 0.01), and the glucose level in DM + Ex-4 group was decreased obviously compared to that of DM group (*P* < 0.01) (Table 1).

### 3.2. Insulin Sensitivity

The whole-body insulin sensitivity was assessed by GIR. According to our previous study [13], high-fat diet feeding for 10 weeks may induce significant insulin resistance in wistar rats. So in this study the rats were given high-fat diet feeding for 10 weeks and then given STZ intraperitoneal injection to conduct T2DM model. At the end of study, the GIR of IR group and DM group was (4.9 ± 0.4) mg∗kg^−1^∗min⁡^−1^ and 4.7 ± 0.4 mg∗kg^−1^∗min⁡^−1^, respectively, both being significantly lower than that of NC group [(7.2 ± 0.5) mg∗kg^−1^∗min⁡^−1^] (all *P* < 0.01). After the intervention with Ex-4, the GIR in IR + Ex-4 group and DM + Ex-4 group were both increased significantly in comparison with that of IR and DM group accordingly (all *P* < 0.01) (Table 1).

### 3.3. CTRP3 mRNA Expression at the Different Stages of T2DM Pathogenesis

**Figure 2:**
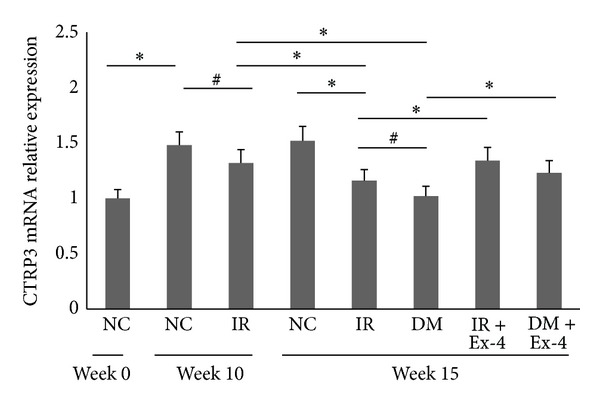
CTRP3 mRNA expression at the different stages of T2DM pathogenesis. NC: normal control group; IR: insulin resistance group; DM: diabetes mellitus group; IR + Ex-4: insulin resistance plus Exendin-4 treatment group; DM + Ex-4: diabetes mellitus plus Exendin-4 treatment group; ^∗^ *P* < 0.01, ^#^ *P* < 0.05.

The relative expression of CTRP3 mRNA in NC group at week 10 was increased significantly than that of week 0 (*P* < 0.01), but there was no significant different between week 10 and week 15. CTRP3 mRNA relative expression of IR group at week 15 was decreased significantly by 12.1% (*P* < 0.01) than that of week 10 which was lower statistically than that of NC group at the same week (*P* < 0.05). Compared to IR group at week 10, the CTRP3 mRNA relative expression in DM group at week 15 was decreased by 22.7% (*P* < 0.01). At week 15, the CTRP3 mRNA relative expression in IR group and DM group was decreased by 25.0% and 32.9%, respectively, in comparison with that of NC group (all *P* < 0.01). The difference of CTRP3 mRNA expressions in DM group and IR group at week 15 was also significant (*P* < 0.05) (Figure 2).

### 3.4. CTRP3 Protein Expression at the Different Stages of T2DM Pathogenesis

**Figure 3:**
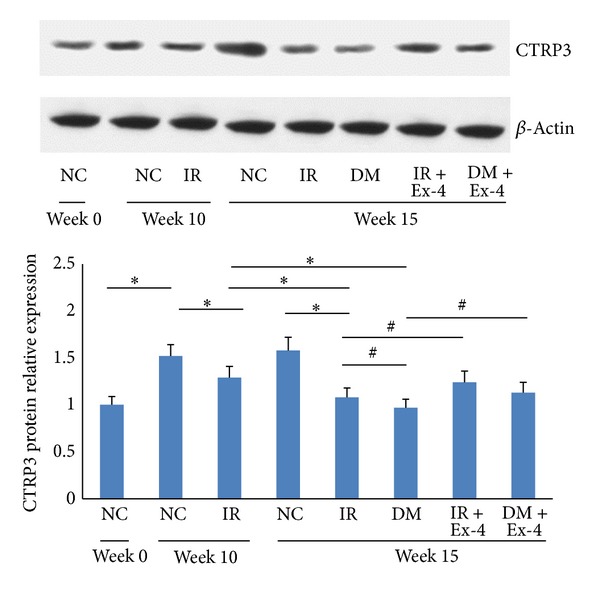
CTRP3 protein expression at the different stages of T2DM pathogenesis. NC: normal control group; IR: insulin resistance group; DM: diabetes mellitus group; IR + Ex-4: insulin resistance plus Exendin-4 treatment group; DM + Ex-4: diabetes mellitus plus Exendin-4 treatment group; ^∗^ *P* < 0.01, ^#^ *P* < 0.05.

Compared with NC group at week 0, the relative expression of CTRP3 protein in NC group at week 10 was increased significantly (*P* < 0.01). There was no significant difference in CTRP3 protein expression of NC group between week 10 and week 15. CTRP3 protein relative expression of IR group at week 15 was decreased significantly by 16.3% (*P* < 0.01) than that of week 10 which was lower statistically than that of NC group at the same time (*P* < 0.01). Compared to IR group at week 10, the CTRP3 protein relative expression in DM group at week 15 was decreased by 24.8% (*P* < 0.01). Compared to NC group at week 15, the CTRP3 protein relative expression of IR group and DM group was decreased by 31.6% and 38.6%, respectively (all *P* < 0.01). The difference of CTRP3 protein expression in DM group and IR group at week 15 was also significant (*P* < 0.05) (Figure 3).

### 3.5. Effects of Ex-4 on the Expression of CTRP3 in T2DM and IR Groups

Compared to IR group at week 15, the relative expression of CTRP3 mRNA and protein of IR + Ex-4 group was increased by 15.5% (*P* < 0.01) and 14.8% (*P* < 0.05), respectively. The relative expression of CTRP3 mRNA and protein of DM + Ex-4 group was increased by 20.6% (*P* < 0.01) and 16.5% (*P* < 0.05), respectively, in comparison with that of DM group (Figures 2-3).

## 4. Discussion

Nowadays there are numerous animal models available for the study of T2DM, but the pattern of disease development and progress in most of them is not equal to the clinical situation in human beings [14]. Many studies have shown that rats fed with high-fat diet develop insulin resistance but not frank hyperglycemia or diabetes, indicating that high-fat diet might be a good way to initiate insulin resistance which is one of the important features and motivators of T2DM [15]. STZ is widely used to reproducibly induce diabetes mellitus by inducing pancreatic islet beta cells death through DNA alkylation. It is well known that high-dose STZ severely impairs insulin secretion, mimicking type 1 diabetes mellitus, and low-dose STZ after high-fat diet feeding induces a mild damage to beta cells on the background of insulin resistance, which is similar to the features of T2DM pathogenesis [16].

Our laboratory has used high-fat diet to induce insulin resistance model and high-fat diet plus low-dose STZ intraperitoneal injection to conduct T2DM model in wistar rats [13, 17]. In this study, male wistar rats were fed with high-fat diet for 10 weeks to induce insulin resistance and then 22 of them were given low-dose STZ (25 mg/Kg) intraperitoneal injection. One week later, the random glucose was detected and there were 15 rats whose glucose was ≥16.7 mmol/L twice not in one day. The diabetes model success rate was 68.2%, similar to other reports [18]. In addition to the detection of glucose, this study also measured GIR though hyperinsulinemic euglycemic clamp to reflect the insulin sensitivity, and found that the GIR of IR group and DM group was lower significantly than that of NC group. Based on the results of glucose and GIR, week 10 (rats fed with high-fat diet for 10 weeks) and week 15 (rats fed with high-fat diet plus low-dose STZ intraperitoneal injection) were considered as different stages of T2DM pathogenesis, that is insulin resistance and overt diabetes.

Recently, CTRP3 was described as a novel adipokine with glucose-lowering effects achieved by suppression of hepatic gluconeogenesis [5]. Schmid et al. [7] used human subcutaneous and visceral adipocytes and murine 3T3-L1 adipocytes to analysis of CTRP-3 expression and function and found that CTRP-3 was expressed in subcutaneous and visceral adipocytes, being positively regulated by insulin and negatively by infection or inflammation-related factors. One study on women with PCOS which is associated with obesity, insulin resistance, and diabetes reported that the levels of serum and omental adipose tissue CTRP3 were lower in women with PCOS in comparison with control subjects [9]. However, another study found that CTRP-3 concentrations were significantly higher in patients with T2DM or pre-diabetes than the normal glucose tolerance group [8]. The possible explanations for the diverges between the two studies might be the effects of medications taken by T2DM patients, which may increase the expression of CTRP3, and the different ethnic groups. Although there have been some researches about the expression of CTRP3 under different status, the pattern of CTRP3 expression and its regulation at the different stages of T2DM is widely unknown.

This study detected the mRNA and protein expression levels of CTRP3 in T2DM rats at different pathogenic stages. In normal control rats, the expression of CTRP3 mRNA and protein at week 10 (aged 20 weeks) was increased significantly than that of week 0 (aged 10 weeks). Although the expressions of CTRP3 mRNA and protein were increased at week 15 (aged 25 weeks) in comparison with week 10, the differences were not significant, indicating that the expression of CTRP3 in visceral adipose tissues of normal rats increased with their growth and came to a relative stable state at the age of 20 weeks. As to T2DM rats, the expressions of CTRP3 mRNA and protein at week 10 when they were at the stage of insulin resistance were lower than that of normal control rats at the same week, and decreased more at week 15 when they were at the stage of overt diabetes. To our knowledge, this is the first time to report the expression of CTRP3 in rats at different stages of T2DM pathogenesis. These results resembled a report of CTRP3 expression in PCOS women as mentioned above but were opposite to one study showing that CTRP3 expression was decreased in T2DM patients in comparison with normal control subjects. The reasons may be the different species, the possible therapies in T2DM patients, and other unknown factors.

It is well known that inflammation is the common characteristic and mechanism of obesity, insulin resistance and T2DM [19]. Our former studies in insulin resistance rats induced by high-fat diet and T2DM rats induced by high-fat diet plus low-dose STZ intraperitoneal injection found that there were obvious inflammation in such animals which were the same as that used in this study [20, 21]. As some studies reported that CTRP3 expression in adipocytes were positively regulated by insulin and negatively by infection or inflammation-related factors, it is reasonable to infer that the relative low expression of CTRP3 in insulin resistance rats and T2DM rats was due to the status of inflammation and insulin resistance in them.

From recent decades, increasing the GLP-1 activity has emerged as a useful therapeutic tool for the treatment of T2DM [22]. The actions of GLP-1 on pancreatic islet beta cells and central nervous and digestive systems have been widely studied [23]. The action of this peptide in adipose tissue, however, is still poorly defined. Studies have shown the presence of GLP-1 receptor in adipose tissue, and that the expression of GLP-1 receptor mRNA and protein was increased in visceral adipose depots from morbidly obese patients with a high degree of insulin resistance [24]. Furthermore, prospective studies carried out with patients that underwent biliopancreatic diversion surgery showed that subjects with high levels of GLP-1 receptor expression in adipose tissue, which indicates a deficit of GLP-1 in this tissue, were those whose insulin sensitivity improved after surgery, suggesting the potential relationship between GLP-1 activity and insulin sensitivity [24]. This study found that after the treatment with Ex-4 for 4 weeks, GIR of IR rats and T2DM rats were increased significantly, indicating the sensitization of GLP-1 in adipose tissue, which may be due to its activities in inhibiting inflammatory and regulating adipogenesis and lipid metabolism [25, 26].

For the tight relationship between GLP-1 and adiposity and its related diseases, more and more studies focused on the effects and mechanisms of GLP-1 on adipose tissue which is the main source and target of adipokines that may be the key modulators in the pathogenesis of adiposity related diseases. GLP-1 receptor agonist has been shown to increase the expression of such adipokines as adiponectin [12] and visfatin [27] that may increase the insulin sensitivity in adipose tissue. CTRP3 is a novel multifunctional adipokine and its expression modulated by GLP-1 has never been studied before. This study, for the first time, reported that Ex-4 treatment increased the mRNA and protein expressions of CTRP3 in insulin resistance rats and T2DM rats. Kopp et al. [28] reported that the recombinant CTRP3 dose-dependently inhibited the release of chemokines in monocytes and adipocytes* in vitro* and* ex vivo*, and inhibited monocyte chemoattractant protein-1 (MCP-1) release in adipocytes, whereas small interfering RNA (Si-RNA)-mediated knockdown of CTRP-3 upregulated MCP-1 release, reduced lipid droplet size, and decreased intracellular triglyceride concentration in adipocytes, causing a dedifferentiation into a more proinflammatory and immature phenotype. These results indicated that CTRP3 has the effects of anti-inflammation, which may be one of the indirect mechanisms that GLP-1 increased insulin sensitivity in insulin resistance rats and T2DM rats as found in this study.

In whole, this study found that the mRNA and protein expressions of CTRP3 in visceral adipose tissue were progressively decreased at the pathogenic stages of insulin resistance and overt diabetes in a T2DM rats model induced by high-fat diet plus low-dose STZ intraperitoneal injection, while Ex-4, a GLP-1 receptor agonist, increased the expression of CTRP3 in such animals and improved their insulin sensitivity. But the expression of CTRP3 at different stages of T2DM pathogenesis in human beings, the factors that modulates the expression of CTRP3 in the pathogenesis of T2DM and the mechanisms that GLP-1 receptor agonists modulate the expression of CTRP3 are still largely unknown and need further research.

## Figures and Tables

**Table 1:** Body weight, FBG, and insulin sensitivity of rats at the end of the study (mean ± SE).

Group	*N*	Body weight (g)	FBG (mmol/L)	GIR (mg ∗ kg^−1^ ∗ min^−1^)
NC	8	410.3 ± 23.6	5.1 ± 0.6	7.2 ± 0.5
IR	8	516.5 ± 30.7*	5.9 ± 1.1	4.9 ± 0.4*
DM	7	485.6 ± 26.8^∗◊^	13.2 ± 2.1^∗Δ^	4.7 ± 0.4*
IR + Ex-4	8	446.9 ± 25.6^#Δ□^	5.2 ± 0.8^□^	5.9 ± 0.5^∗Δ□^
DM + Ex-4	8	432.2 ± 24.7^Δ□^	7.6 ± 0.9^∗Δ□^	5.8 ± 0.5^∗Δ□^

NC: normal control group; IR: insulin resistance group; DM: diabetes mellitus group; IR + Ex-4: insulin resistance plus Exendin-4 treatment group; DM + Ex-4: diabetes mellitus plus Exendin-4 treatment group; FBG: fasting blood glucose; GIR: glucose infusion rate; versus NC group *P < 0.01, ^#^ *P* < 0.05; versus IR group ^Δ^ *P* < 0.01, ^◊^ *P* < 0.05; versus DM group ^□^ *P* < 0.01.
